# Development of EST-SSR markers based on transcriptome and its validation in ginger (*Zingiber officinale* Rosc.)

**DOI:** 10.1371/journal.pone.0259146

**Published:** 2021-10-27

**Authors:** Venugopal Vidya, Duraisamy Prasath, Mohandas Snigdha, Ramasamy Gobu, Charles Sona, Chandan Suravi Maiti

**Affiliations:** 1 ICAR-Indian Institute of Spices Research, Kozhikode, Kerala, India; 2 School of Agricultural Sciences and Rural Development (SASARD), Nagaland University, Nagaland, India; Siksha O Anusandhan University, INDIA

## Abstract

Ginger (*Zingiber officinale* Rosc.) is an economically important and valuable spice crop around the world. It is used as food, spice, condiment, and medicine. A considerable extent of genetic diversity in ginger occurs in the Western Ghats and North-Eastern India. However, genetic diversity studies at the molecular level in ginger is limited due to limited availability of genetic and genomic information. In the present study, for the first time, we have identified and validated expressed sequence tag (EST)-simple sequence repeat (SSR) markers from ginger. We obtained 16,790 EST-SSR loci from 78987 unigenes, and 4597 SSR loci in the predicted 76929 coding sequences from RNA-Seq assembled contigs of ginger through Illumina paired-end sequencing. Gene ontology results indicate that the unigenes with SSR loci participate in various biological processes such as metabolism, growth, and development in ginger. One hundred and twenty-five primer pairs were designed from unigenes and coding sequences. These primers were tested for PCR optimization, characterization, and amplification and identified 12 novel EST-SSR markers. Twelve flanking polymorphic EST-SSR primers were validated using 48 ginger genotypes representing North-Eastern India and different eco-geographical adaptations by PCR amplification and allele sizing through capillary electrophoresis. Twelve EST-SSR primers generated a total of 111 alleles with an average of 9.25 alleles per locus and allele sizes ranging between 115-189bp. This study implies that the SSR markers designed from transcriptome sequences provides ample EST-SSR resources, which are helpful for genetic diversity analysis of Zingiberaceae species and molecular verification of ginger genotypes.

## Introduction

Ginger (*Zingiber officinale* Rosc.), an important spice and medicinal plant is known since antiquity. It belongs to the family Zingiberaceae and has various medicinal properties such as antioxidant, antimicrobial, anti-diabetic, and anti-inflammatory effects [1]. It is believed to be originated in South East Asia [2]. A wide array of diversity occurs in the Western Ghats, India [3, 4], and maximum variation within cultivated ginger occurs in North-Eastern India. However, less genomic information and limited molecular markers availability in ginger hinders breeding and genetic studies.

To conserve the genetic resources and achieve improved crop productivity, it is essential to understand genetic diversity [5]. Due to the quick advancement in molecular biology, progress in DNA based markers was growing rapidly [6]. Microsatellites, also known as simple sequence repeats, are co-dominant DNA markers of short tandem repeats of 2 to 6 bp of nucleotides that are extensively present in whole genomes of various species. Microsatellites are of two types viz, genomic SSR and expressed-sequence-tag-based/ genic SSR (EST-SSR). Being co-dominant in nature, SSR markers have an edge over other markers, such as, high level of polymorphism, high specificity and repeatability [7]. Hence, SSR markers are widely used to study germplasm characterization, QTL mapping, linkage mapping and marker-assisted selection (MAS) [8].

With minimal molecular information available for ginger, molecular characterization was attempted earlier by using ISSR [9, 10], AFLP [11], RAPD [12] and SSR [13, 14] molecular markers. Since the EST-SSRs tightly linked to the genes controlling different traits can reveal local adaptation and environmental heterogeneity compared to other neutral markers [15, 16]. Besides that, it can be very efficiently transferred to other related species even if they are designed for a specific crop because of their location in conserved regions [17–19]. With improvements in next-generation sequencing (NGS) technology, such as low cost *de novo* transcriptome sequencing, has facilitated the identification of microsatellite loci [20]. The *de novo* assembly of transcriptomes is requisite for analysing functional genomics or marker development in non-model organisms, especially when there is a lack of a sequenced genome [20, 21].

Previously, we sequenced the transcriptome of ginger [22] using Illumina sequencing. In this study, we assembled 78987 unigenes, identified EST-SSR loci, designed primer pairs based on these data. The primary goal of this study was to develop EST-SSR markers from transcriptome sequence for the genetic diversity study among ginger genotypes and to preserve their diversity. In addition, these markers may be helpful in the ginger taxonomic, and evolutionary history research.

## Materials and methods

### *De novo* assembly and functional annotation of the transcriptome

The ginger transcriptome was sequenced earlier using Illumina sequencing [22]. The raw reads of this transcriptome sequence (PRJNA311170) were initially processed to ensure the accuracy of *de novo* assembly and subsequent analyses of SSR. Fast-QC (v0.11.7) (http://www.bioinformatics.babraham.ac.uk/projects/fastqc/) was used to control raw reads by filtering the empty reads, the adaptor sequences, and the sequences with low quality. Further filtering and quality control of the reads was done with Trimmomatic v.0.32 with default settings [23]. Finally, *de novo* assembly of the transcripts was completed using Trinity Assembler (https://github.com/trinityrnaseq/trinityrnaseq/releases) with default k-mer parameter (K = 25), and contigs were obtained by assembling overlap information between the sequences. After the assembly, unigene sequences were developed by CD-HIT (http://weizhongli-lab.org/cd-hit/) and using the unigene data coding regions were extracted using TransDecoder (https://github.com/TransDecoder/TransDecoder/releases).

The functional annotation of the non-redundant unigenes were done by Blastx against NCBI non redundant protein database and GO annotation and then plotted with functional classification using the BLAST2GO module of OmicsBox programme (www.biobam.com/omicsbox).

### Development of EST-SSR markers

EST-SSR from the unigenes and CDS were mined using MIcroSAtellite identification tool (MISA, http://pgrc.ipk-gatersleben.de/misa/misa.html). The repeat sequence motifs included mono-, di-, tri-, tetra-, penta-, and hexa-nucleotides with a minimum repeat number of 10, 6, 5, 5, 5, and 5, respectively. Batch Primer 3, an online web tool, was used to design SSR primer pairs from the flanking sequences of the identified microsatellite motifs [24]. Primers were designed considering the following conditions (a) primer length of 18 to 25 bp with 20 bp as the optimum; (b) PCR product size ranging from 100 to 300 bp; (c) melting temperature (Tm) between 55°C and 65°C with a difference of no greater than 5°C between the Tm values of the forward and reverse primers; and (d) GC content of 40% to 70% with an optimum of 50%. The designed primer quality was verified using NetPrimer (http://www.premierbiosoft.com/netprimer/index.html). For this study, a total of 125 primers were selected randomly and synthesized by Integrated DNA Technologies (IDT, Coralville, IA).

### Plant material and DNA isolation

A total of 48 ginger genotypes, which includes 27 landraces of the North-Eastern States of India, 19 cultivated genotypes, one exotic accession, and *Zingiber zerumbet* (S1 Table) were collected from the National Active Germplasm Site of ginger at ICAR-IISR, Experimental Farm, Kozhikode, Kerala, India. The collected rhizomes were planted, and young disease-free leaves were collected from the 48 genotypes. Genomic DNA was isolated from the fresh leaf samples using Doyle and Doyle [25] with modifications such as double centrifugation with chloroform isoamyl alcohol (24:1) to get a clear supernatant free from polyphenols. The DNA was dissolved in 50 μL of nuclease-free water. The quantity and quality of DNA samples were determined using the DeNovix DS-11 spectrophotometer (DeNovix, Wilmington, DE, USA) and by electrophoresis on 0.8% agarose gel, then diluted to 50 ng/μL and stored at − 20°C until the PCR analysis.

### EST-SSR markers amplification and validation

A set of 125 EST-SSR primers designed randomly to screen 48 ginger genotypes for validating SSR locus using PCR amplification (Agilent Surecycler 8800, USA). A total of 20 μl PCR reaction mix contained 10 μl of PCR master mix (Emerald Amp GT PCR, Takara), 0.5 μl (10 μM) each forward and reverse primers, 1 μl template genomic DNA (50ng/ μl), and 8 μl nuclease-free water. PCR amplification was done under the following conditions: initial denaturation of template DNA at 94°C for 5 min followed by 35 cycles of 94°C for 45 s, 50 to 65°C (depending on the melting temperature of the primer pair used) for 45 s and 72°C for 1 min followed by a final extension at 72°C for 10 min. PCR products were separated on 3.5% agarose gel, stained with ethidium bromide, and gels were visualized using a gel documentation system (Syngene Gel Doc; Syngene, Synoptics Ltd, UK). After analyzing the agarose gel, 12 primers that showed polymorphism were further selected for capillary electrophoresis. The amplified PCR products of the selected primers were loaded into a QIAxcel capillary gel electrophoresis system (QIAGEN, Germany) to confirm SSRs in amplified genomic DNA fragments. The allelic sizes of each sample were measured in the form of virtual gel images and electropherogram peaks using QIAxcel Screengel Software (QIAGEN, v1.5).

### Data processing and genetic analysis

Reproducible and consistent SSR bands were scored as present (1) or absent (0) into a binary matrix separately for the 48 ginger genotypes. The polymorphic information content (PIC) of selected SSR primers was calculated by the formula: PIC = 1−Σ(Pi)^2^, where Pi is the frequency for the i^th^ microsatellite allele. The genetic similarity (GS) among the genotypes was calculated by Jaccard’s similarity coefficients. The dendrogram was constructed through SAHN clustering method, and similarity coefficients generated using the unweighted pair group method with arithmetic mean (UPGMA) in NTSYS-pc version 2.01 [26]. Principal coordinate analysis (PCoA) based on the simple matching coefficient using eigen vector matrices was performed to confirm the grouping in the software GenAlex version 6.5.

## Results

### Identification of frequency and distribution of EST-SSRs

RNA sequences of the ginger reported in a previous study [22] were assembled to form contigs. A total number of 78987 contigs were generated from 145942 transcripts with an average contig length of 840.72 bp. The N50 value was 1,099 and the GC content was 44.79%. The median contig length was 574 bp. The predicted 78987 unigene contains both coding and non-coding sequences (Tables 1 and 2). TransDecoder identified long open reading frames (ORFs) within transcripts and scores them according to their sequence composition. The predicted coding regions were 76929.

**Table 1:** Summary of assembly statistics of the ginger transcriptome data.

Total contigs	79049
Total transcripts	193666
GC (%)	44.7
N50	1236
Median contig length (bp)	629
Average contig length (bp)	912.83
Total assembled bases	176783582

**Table 2:** Frequency distribution of the 12 most frequent EST-SSR repeat motifs in ginger.

Repeats	5	6	7	8	9	10	11	12	13	14	15	>16	Total
A/T	-	-	-	-	-	4027	1749	890	610	395	266	527	8464
AG/CT	-	800	473	297	194	154	103	90	23	39	43	70	2286
AGG/CCT	599	283	167	92	20	22	1	5		2		0	1191
AAG/CTT	623	230	127	66	18	29	7	7	1	3		1	1112
CCG/CGG	457	196	74	31	21	6						0	785
AT/AT	-	228	107	73	50	25	23	10	4	8	1	1	530
AGC/CTG	304	128	52	32	1	1	5					0	523
AC/GT	-	145	66	44	21	23	13	10	8	2	2	8	342
ATC/ATG	170	36	13	25	3	12	1					0	260
AAC/GTT	106	69	22	11	1	6	1					0	216
ACG/CGT	100	38	33	16	8							0	195
ACC/GGT	97	51	15	5	2							0	170
AAT/ATT	74	32	18	18	4	1		2				0	149
C/G	-	-	-	-	-	46	19	8	8	5	2	2	90

**Fig 1:**
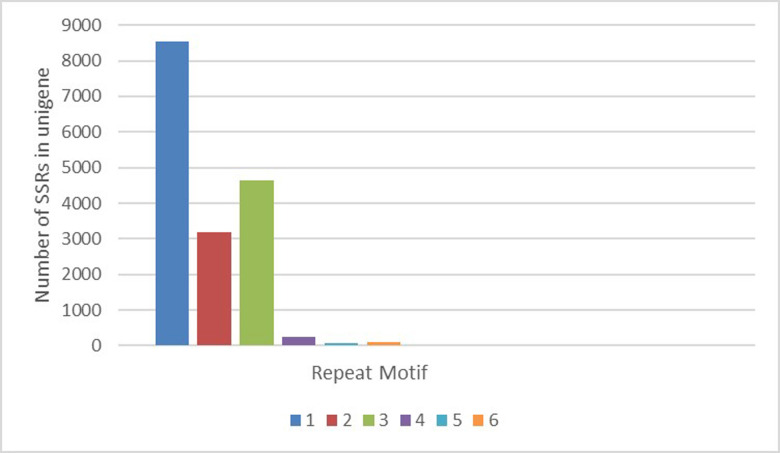
Distribution to different repeat type classes in unigenes.

**Fig 2:**
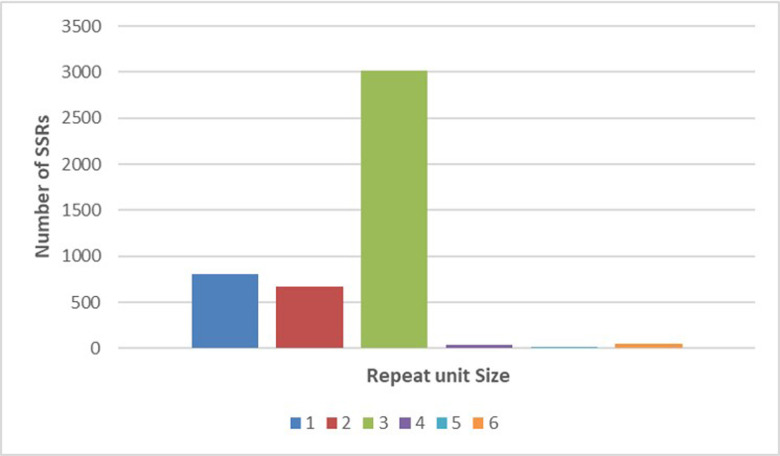
Distribution to different repeat type classes in CDS.

A total of 16790 SSRs were identified using MISA software in 145942 sequences, with 1742 unigenes containing more than one SSR locus. Additionally, 710 SSRs were involved in compound formation. The SSR repeats units ranged from one to six, and the number of SSRs with each repeat motif varied widely. Among the SSRs, mono-nucleotide repeat motifs were found most abundant (8554; 50.94%), followed by tri- (4639; 27.62%), di- (3176; 18.91%), tetra- (252; 1.50%), hexa (101; 0.61%), and penta- (68; 0.40%) repeat motifs. Among the two types of molecular mononucleotide repeats (A/T) n was the most abundant when compared to (C/G) (Fig 1). While 4597 SSRs were identified in the predicted 76929 coding sequences, 334 sequences containing more than one SSR locus were identified. Additionally, 191 SSRs involved in compound formation. SSRs with trinucleotide repeat motifs were most abundant (3020; 65.6%), followed by mono (809; 17.6%), di- (666; 14.48%), hexa- (48; 1.04%), tetra (41; 0.89%), and penta- (13; 0.28%) repeat motifs (Fig 2).

#### Functional annotation and classification of unigenes

**Fig 3:**
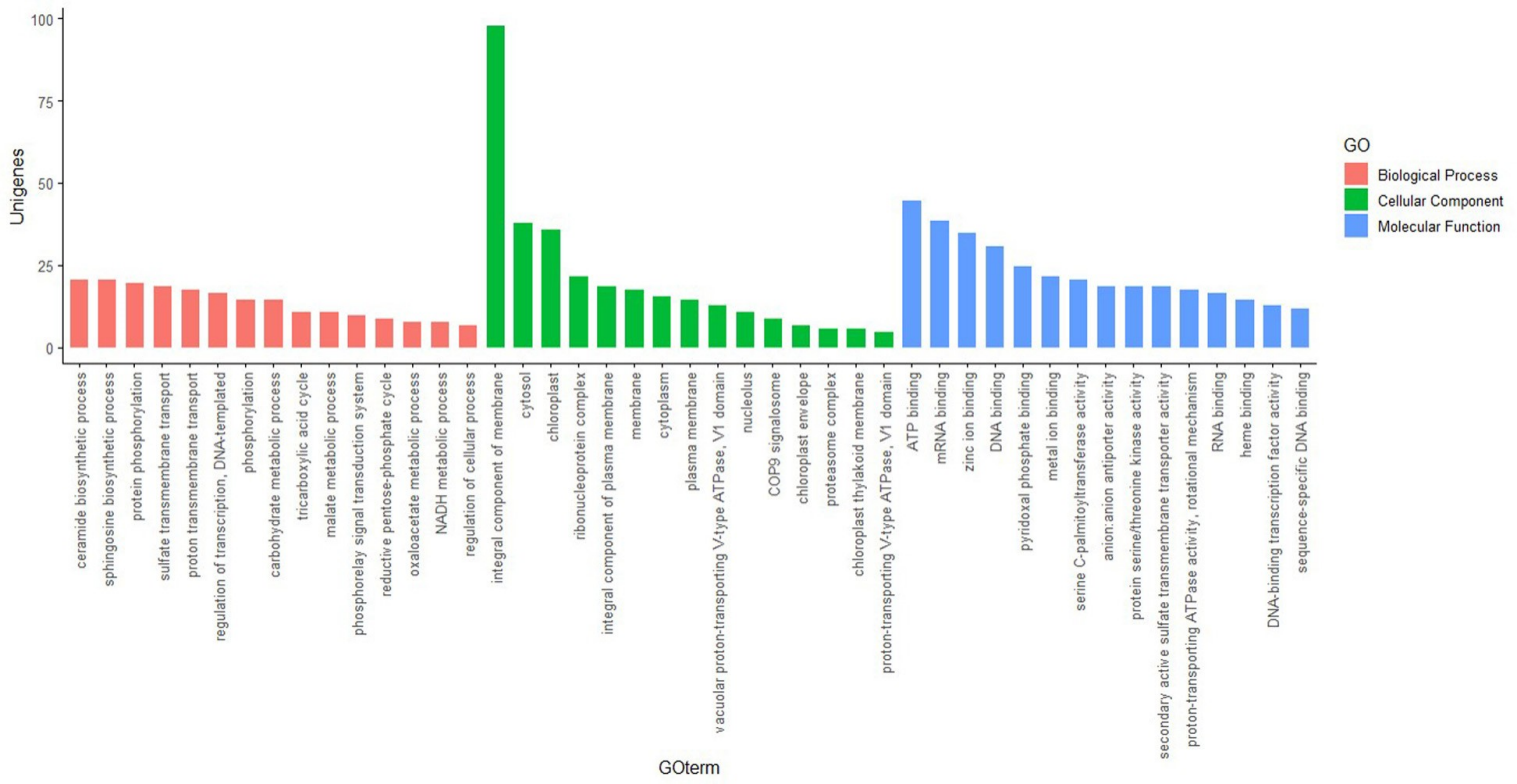
Gene Ontology classification of unigenes. Unigenes were assigned to three categories: cellular component, molecular function, and biological process.

The unigenes were functionally annotated using BLAST2GO module of OmicsBox. 18222 unigenes were mapped to GO database. The sequence length of the annotated unigenes ranges from 301 to 14178 nucleotides. 10269 unigenes were mapped to known enzymes with EC numbers. 15965 unigenes had similarity measures >70%. The E-value distribution ranged from 0 to 9.85E-11. The annotated unigenes were grouped into three clusters: molecular function, biological process, and cellular component (Fig 3 and S2 Table). The cluster biological process consisted of maximum number of GO terms (1951) whereas cellular component consisted of least (506). Molecular Function component included 1407 terms. Majority of the unigenes in biological process were distributed in sphingosine and ceramide biosynthetic pathways. In molecular function, it was chiefly distributed in ATP binding, mRNA binding and zinc ion binding. A high proportion of unigenes were in the cellular component of membrane proteins.

### Development and validation of the SSR markers

**Fig 4:**
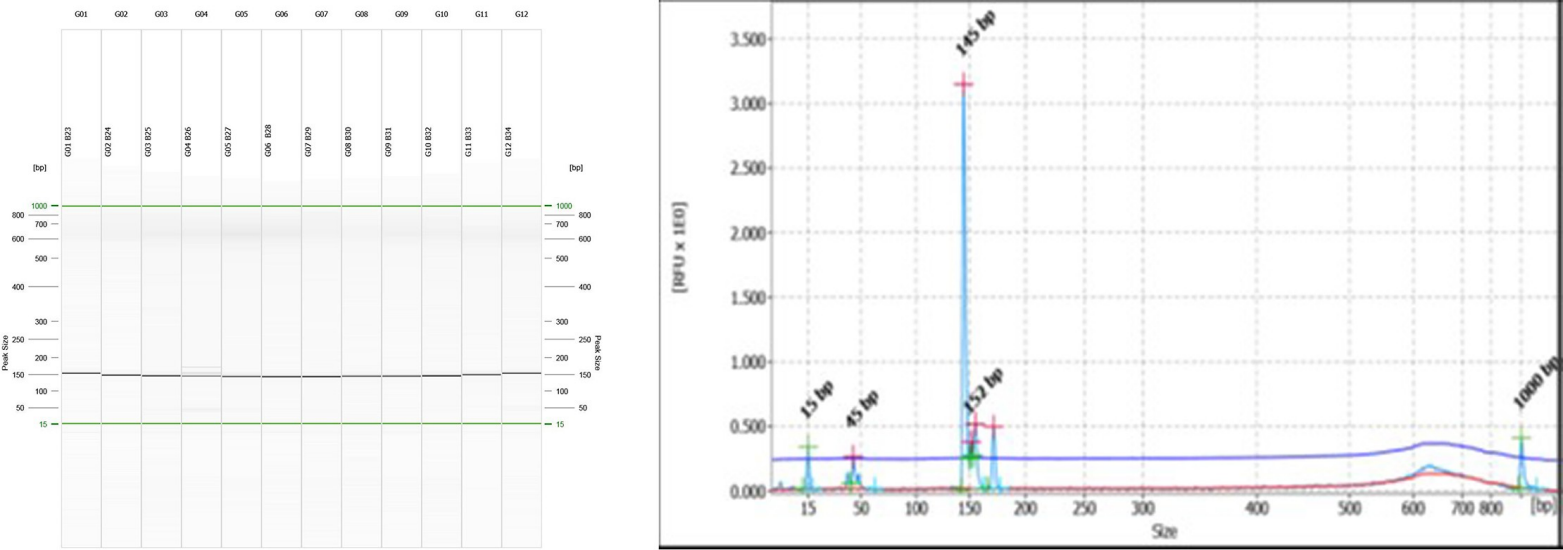
Validation of SSR markers in germplasm accessions of ginger. A. Gel image of PCR amplification of SSR marker ZOSSR25 on 48 germplasm accessions of ginger as captured on QIAxcel ScreenGel software. Numbers refer to accession numbers as indicated in S1 Table. The last lane is DNA molecular weight standard 50-1000bp v2.0 Qx DNA size marker. B. A representative electropherogram showing different allele sizes (sample G04 B26, variety Athira).

Only the SSRs with di-, tri-, tetra-, penta- and hexa-nucleotide repeats were considered as potential candidates for EST-SSR marker development. 125 primer pairs were designed randomly from the unigenes, and CDS, and was tested for PCR optimization, characterization, and amplification. The validation of the designed EST-SSR primers was undertaken by PCR amplification and separation of the PCR products on a 3.5% agarose gel electrophoresis. Out of 125 primers, 92 primers produced amplicons of the expected size. However, the resolution of the EST-SSR primers was poor as the size of the bands observed on the agarose gel was uniform for most of the genotypes. To understand the allelic variation among the genotypes, we have selected 12 prominent EST SSR primers, which showed polymorphism between north-eastern genotypes, red ginger type genotypes and *Z*. *zerumbet* for capillary electrophoresis to study the allelic difference among the 48 genotypes. The characteristics associated with 12 polymorphic SSR markers are listed in Table 3. In total, 111 alleles were identified with an average of 9.25 alleles per locus varying from 5–15, which generated 1610 amplicons in total. In this study, the PIC values of the selected 12 EST-SSRs ranged from 0.51 to 0.90, with an average of 0.78. The lowest PIC value was observed in ZOSSR38 primer with 5 alleles, while the highest PIC value was obtained for ZOSSR91 with 15 alleles (Table 3 and S1 Fig). The ginger variety, Athira, showed a distinct polymorphic band with ZOSSR25, visible from agarose gel and confirmed with capillary electrophoresis (Fig 4).

**Table 3:** Characteristics of 12 selected primers designed for analysing genetic diversity of ginger and PIC value.

Primers	Repeat motif	Sequence	Total alleles	Monomorphic amplicons	Polymorphic amplicons	Total number of amplicons	Allele size range (bp)	PIC value
**ZO SSR2**	(TTC)7	F-TGATTCCGTATCAACTCCAT	10	0	10	138	117–154	0.81
** **		R-CAAGGAAGACTTCAACTCCAT						
**ZO SSR25**	(AAG)5	F-CTGAGTCCAGTGCTGTATAGG	10	0	10	66	143–178	0.81
** **		R-GTTCTTGCTCGCTAGATCAC						
**ZO SSR16**	(GTGATG)7	F-ATCAAGGAAAAGACCTCAAAG	8	0	8	75	115–163	0.67
** **		R-CATTATCATCGTCTTCCTCTG						
**ZO SSR36**	(GAC)8	F-GAGGACTACTCCGATATGAGC	8	0	8	202	162–189	0.82
** **		R-GGAGTTAGGGTTAGGGATTG						
**ZO SSR21**	(CCT)6	F-TCCTCCTCTTTTCTCCTCTC	6	0	6	123	164–182	0.75
** **		R-GCTAGAAGCGAGGGATTT						
**ZO SSR64**	(GGC)6	F-TCCAGAGGTCTCTCAGCTT	9	0	9	74	122–149	0.75
** **		R-ACTGCGACGACTCAGGTC						
**ZO SSR 111**	(TCT)10	F-CTAAGGGGCTCCTTCTTC	11	0	11	123	124–160	0.89
** **		R-CAGCTGGAAGCAGCTATG						
**ZO SSR108**	(TTTA)5	F-GATCTCCTGCTTGTTATCTCTC	8	0	8	164	117–145	0.8
** **		R-TGTTCTAGGTGTTGTGGAAG						
**ZO SSR35**	(GAG)7	F-GGTCCAAGGTCTTTAAGCAT	12	0	12	186	129–195	0.83
** **		R-ACGAAGACAACGATATCAGC						
**ZO SSR38**	(GAAGGC)5	F-GAAGGAGGCTCTCGAAGT	5	0	5	64	158–188	0.51
** **		R-GCACCTGCTTACAGTTACAAT						
**ZO SSR73**	(CCT)5	F-GCTCTCCCTTCGAAAAAC	9	0	9	144	154–178	0.84
** **		R-GCGTAGGTGCAGAAGTAGTTA						
**ZO SSR91**	(GCA)7	F-CTCCATCCTATCAACTGTCAC	15	0	15	251	126–181	0.9
** **		R-ACATTCTGAAGCTCTTGCAT						
**TOTAL**	** **	** **	**111**	** **	** **	**1610**	** **	**9.39**
**Average**	** **	** **	**9.25**	** **	** **	**134.1667**	** **	**0.78**

### Genetic diversity analysis

The Jaccard’s similarity coefficients ranged between 0.07 and 0.84 (S3 Table). The least similarity coefficient of 0.07 was observed between 9079–9019 and *Z*. *zerumbet*–Sourabh genotypes, while the highest similarity coefficient 0.84 was observed between 9080–9042.

**Fig 5:**
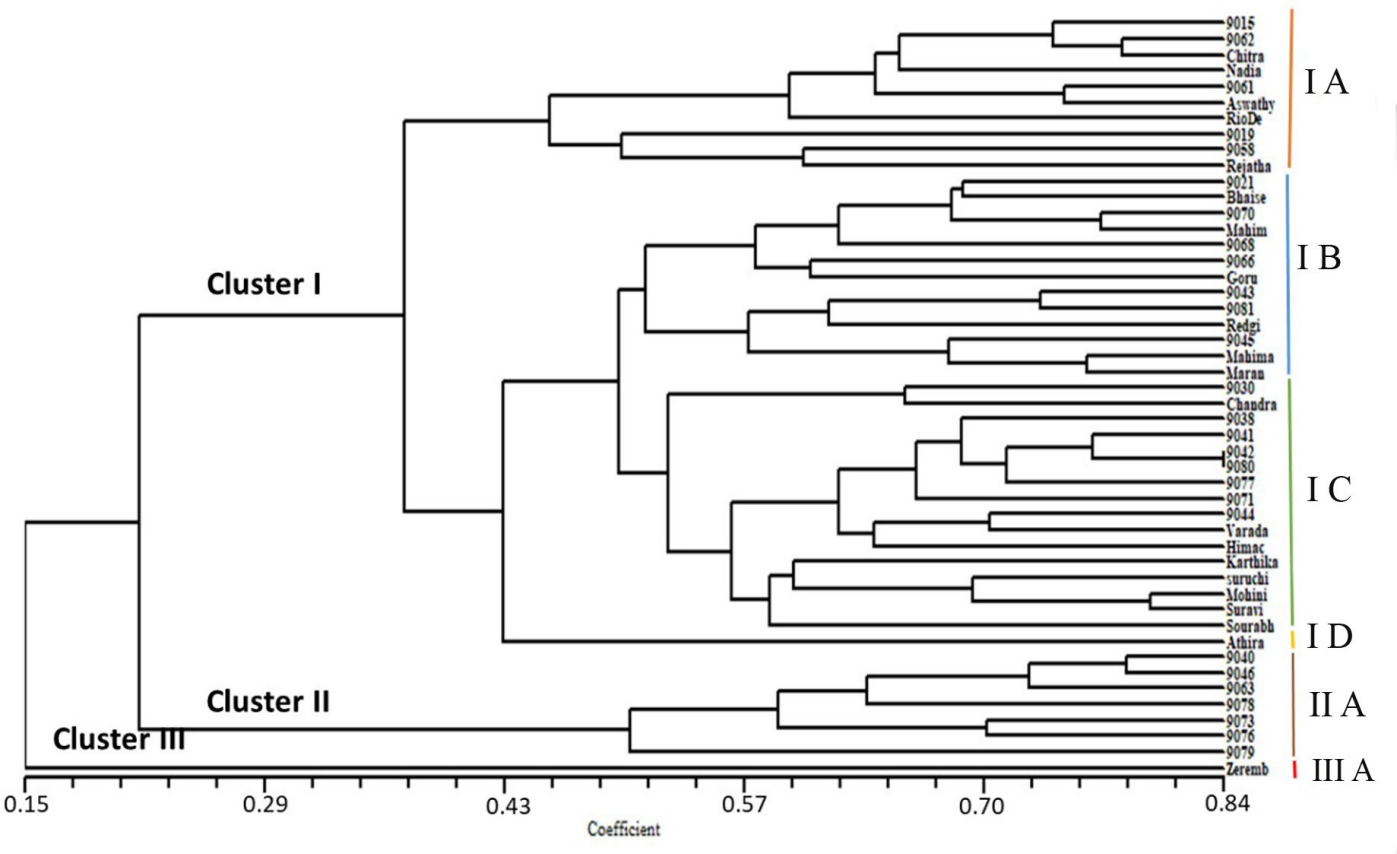
Genetic relationship of ginger accessions as revealed by SSR markers. Dendrogram of 48 ginger genotypes revealed by cluster analysis of genetic similarity estimates generated by Jaccard coefficient based on 12 EST-SSR markers.

**Fig 6:**
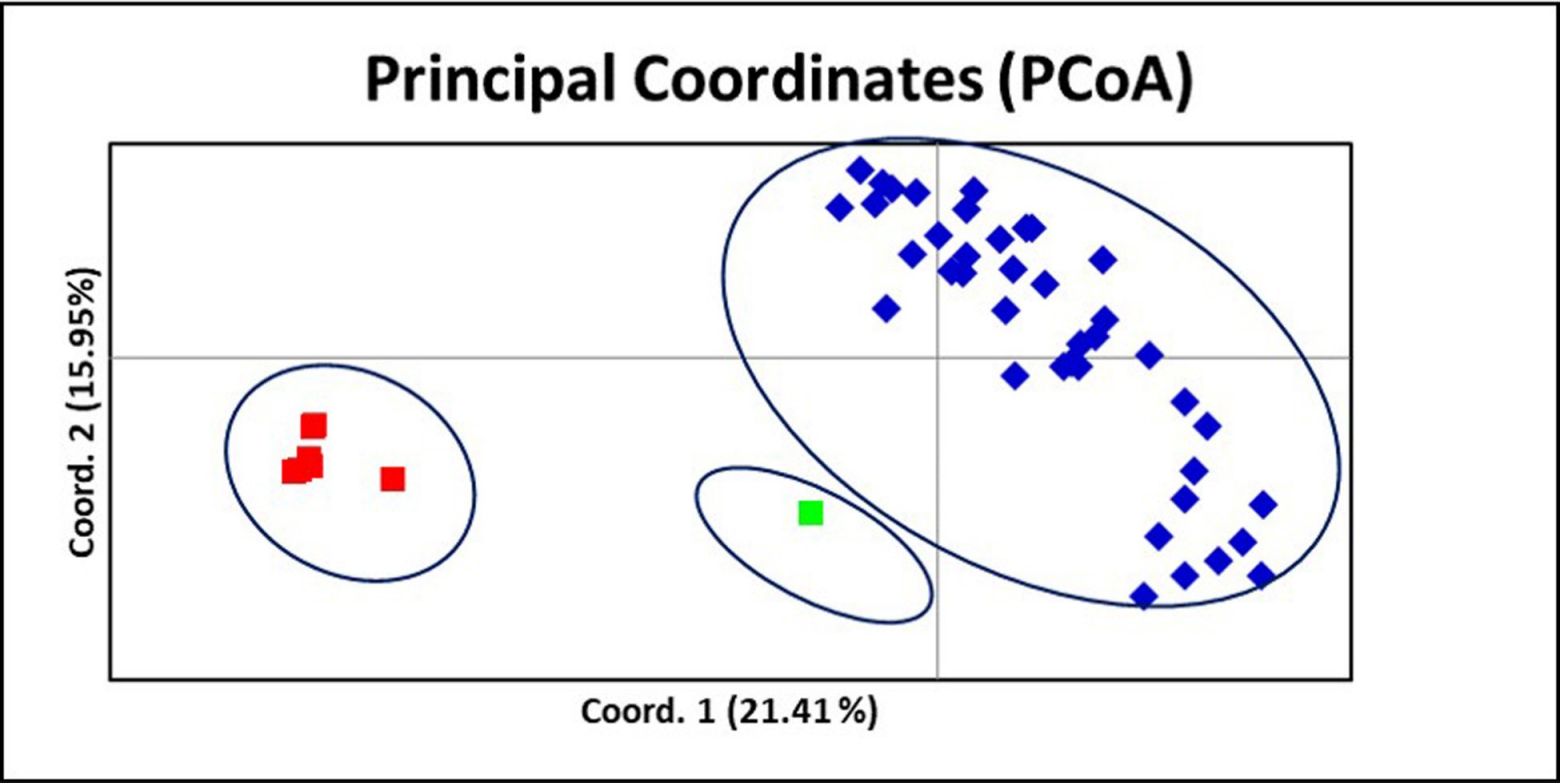
Principle component analysis (PCA) of ginger genotypes based on 12 polymorphic EST-SSR markers.

The UPGMA-based dendrogram placed all 48 genotypes into three main clusters at 15% similarity (Fig 5). Cluster I was the larger cluster and subdivided into four sub-clusters, cluster IA comprised of 10 genotypes (9015, 9062, Chitra, Nadia, 9061, Aswathy, Rio-de, 9019, 9058, Rejatha). Cluster IB comprised of 9021, Bhaise, 9070, Mahim, 9068, 9066, Gorubadhane, 9043, 9081, Exotic Red ginger, 9045, Mahima and Maran. Cluster IC includes 9030, Chandra, 9041, 9042, 9080, 9077, 9071, 9044, Varada, Himachal local, Karthika, Suruchi, Mohini, Suravi and Sourabh and cluster ID has Athira. Cluster II involves all the red ginger type genotypes of the North Eastern Region, India (9040, 9046, 9063, 9078, 9073, 9076, 9079). Cluster III contain *Z*. *zerumbet*. The genetic relationship among the genotypes was further studied using principal coordinate analysis (PCoA), according to the cluster analysis (Fig 6, S4 Table).

## Discussion

### Characterization of EST-SSRs in ginger

Sequence analysis in non-model organisms has been widely studied by *de novo* assembly of short reads of the transcriptome without a reference genome [27, 28]. Because of the high polymorphism, reproducing capacity, and codominance, microsatellite markers are in great demand and widely used in DNA fingerprinting, genetic diversity, population structure analysis, and marker-assisted crop breeding [29, 30]. However, the traditional way of SSR development is a very costly and time-consuming process and hence is not very efficient [31]. In this present study, we have used already available transcriptome data of ginger generated in our lab through Illumina paired-end RNA-seq technology [22]. N50 and an average length of all unigenes was 1,099 bp (Table 2), which is comparable with N50 reported from the *de novo* transcriptome assembly of *Curcuma longa* (N50 = 1,515 bp) [32]; *Zanthoxylum bungeanum* (N50 = 846 bp) [33], *Zantedeschia rehmannii* Engl. (N50 = 1,476 bp) [34], *Cicer arietinum* L. (N50 = 1,192 bp) [35] and *Ipomoea batatas* (N50 = 765 bp) [36]. The functional prediction was performed using Nr and GO protein databases. The unigenes were classified into 45 subcategories, including 15 functions each in molecular functions, biological process, and cellular component aspects (Fig 3). This result is similar with earlier studies on *Triticum aestivum*, *Curcuma alismatifolia* and *Curcuma longa* as the unigenes are classified into 47, 51 and 25 subcategories with GO database respectively [32, 37, 38]. We identified 16,790 EST-SSR loci from 145942 unigenes and 4597 SSR loci identified in the predicted 76929 coding sequences. Trinucleotide repeats were the most frequent SSR repeats when excluding mono repeats followed by di and tetra repeats. Annadurai et al., (2013) [32] in turmeric and Wei et al., (2016) [34] in coloured calla lily reported di and tri-nucleotide motifs as most frequently occurred. In UTRs, a higher percentage of SSRs were mapped than the CDS region mostly due to the long-term natural selection which altered microsatellites that could easily lead to phenotype changes, and SSRs in the non-coding sequences could ensure the stability of germplasm resources [39]. Moreover, in general, EST-SSRs with tri-repeats remained most common among the monocot and dicots because open reading frames do not disturb the triplet codon with insertions and deletions within translated regions [29, 40, 41]. Among mononucleotide repeats, as in most plants, A/T repeats were far more abundant than G/C repeats [42]. Among the di-nucleotide repeats, AG/CT showed prevalence over other di repeats in contrast to Awasthi et al. (2017) [14] where maximum frequencies of TA and GA repeats and low frequencies of other di-nucleotide repeats were reported. The most abundant trinucleotide repeat motif in ginger was AGG/CCT, AAG/CTT and closely followed by CCG/CGG (12.94%); similar results were reported in *Curcuma alismatifolia* [38] and calla lily [34]. Besides, we noticed that, GC rich tri-repeats were more abundant than AT-rich repeats, supporting the fact that GC richness and consequent codon usage bias can be considered specific features of monocot genomes [38].

### Genetic diversity in ginger

A total of 125 primer pairs were designed based on the ginger transcriptome sequences, and 92 primer pairs were successfully amplified. However, the remaining 33 primers did not produce amplicon in the ginger genotypes, indicating the primer either developed from an erroneously assembled transcript or the primer sequence exon-exon junctions [43]. In this study, 12 EST-SSR markers (Table 3) were obtained and verified, offering an informative and applicable approach for evaluating genetic relationships within and among the ginger genotypes. Besides, the PIC value is normally used to quantify polymorphism for a marker locus and is determined by both the number of alleles and their frequency distribution within the population [43]. All the ginger SSRs validated here showed a moderate level of informativeness (PIC > 0.5), with an average PIC value of 0.78.

The present study demonstrated the utility of the 12 newly developed polymorphic EST-SSR markers to evaluate genetic diversity among ginger genotypes. From the analysis results, the 12 markers divided the 48 genotypes into three main groups using the UPGMA cluster analysis. The dendrogram revealed that the North Eastern collections are highly diverse and falls in a different cluster along with other cultivated ginger varieties; also, red ginger type north-eastern collection fell into a single cluster and which is not clustered along with exotic Red ginger indicating the diverse nature of North Eastern red ginger collections as a separate cluster. Previous reports on EST-SSR in ginger focused on cross-species transferability [14, 44]; however, we report 12 novel EST-SSR which can be used for the diversity analysis of wide range of ginger germplasm and landraces.

## Conclusion

The present work constitutes a considerable progress in identifying enormous number of informative SSR loci in ginger from transcriptome sequences. The expressed sequence tag (EST)–SSR markers will be helpful in the future investigation of genetic diversity, population structure, and phylogeography of the species. Furthermore, all the markers were successfully cross amplified in *Z*. *zerumbet* species, suggesting that they may also be used to study other related species in the Zingiberaceae family. Moreover, this study will aid in the development of conservation and management of existing ginger germplasm and endemic landraces unique for a particular geographical location.

## Supporting information

S1 Fig Gel images of SSRs with highest and lowest alleles. Gel image of PCR amplification of SSR marker ZOSSR38 with least alleles on 48 germplasm accessions of ginger as captured on QIAxcel ScreenGel software.
(TIF)

S2 Fig Gel images of SSRs with highest and lowest alleles. Gel image of PCR amplification of SSR marker ZOSSR91 with highest alleles on 48 germplasm accessions of ginger as captured on QIAxcel ScreenGel software.
(TIF)

S1 Table Ginger genotypes used for validation of EST-SSR markers and diverity analysis. (XLSX)

S2 Table Gene ontology annotation of unigenes. (XLSX)

S3 Table Jaccards similarity coefficient. (XLSX)

S4 Table PCoA analysis and Eigen values using GenAlEx software. (XLSX)
