# Supplementary figures and images for: Development of EST-SSR markers based on transcriptome and its validation in ginger (Zingiber officinale Rosc.)
Source: PLoS One. 2021 Oct 27;16(10):e0259146. doi: 10.1371/journal.pone.0259146 (PMC8550423; doi:10.1371/journal.pone.0259146)

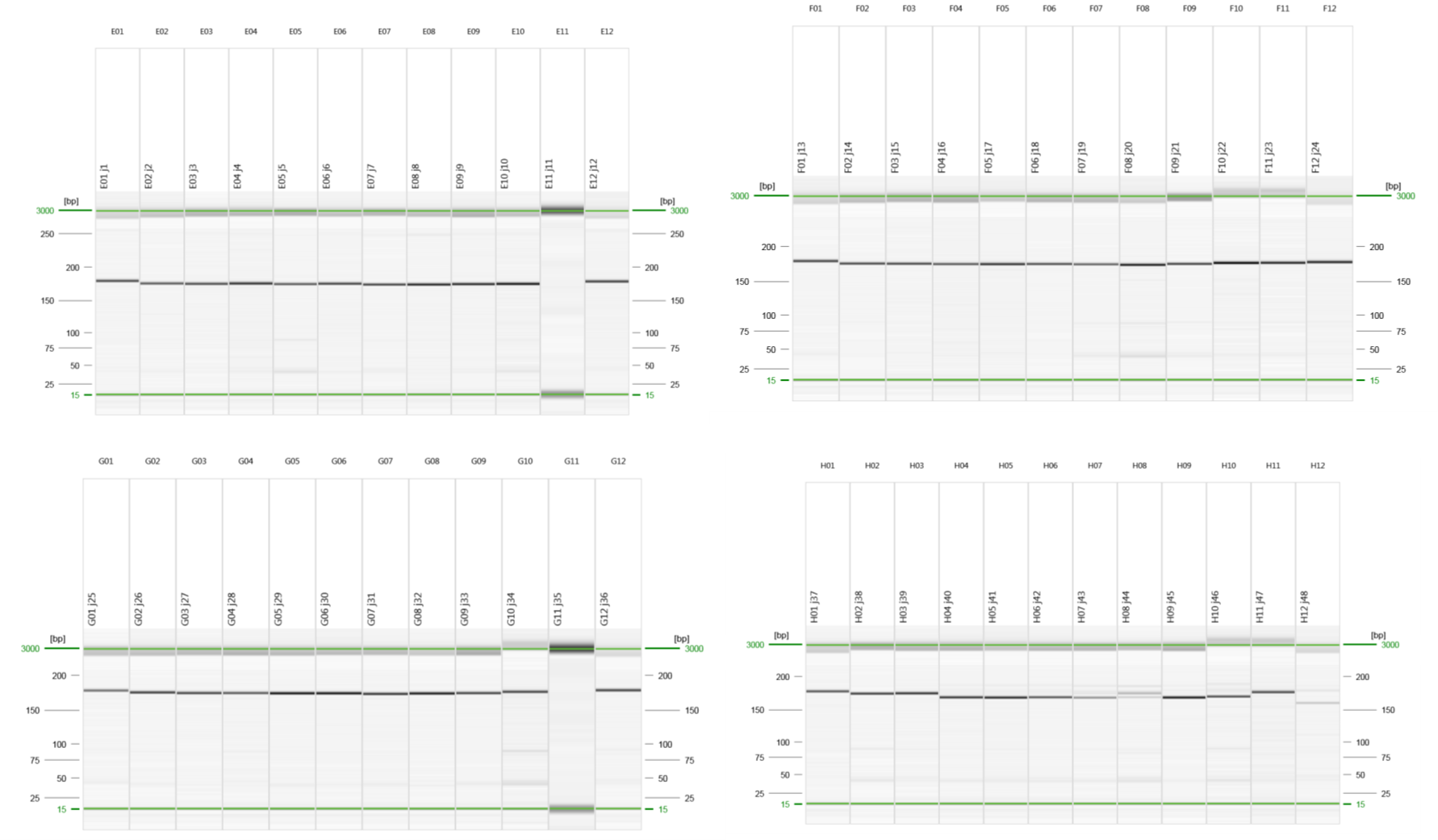

Supplement: S1 Fig — Gel image of PCR amplification of SSR marker ZOSSR38 with least alleles on 48 germplasm accessions of ginger as captured on QIAxcel ScreenGel software. (TIF) [file pone.0259146.s001.tif]

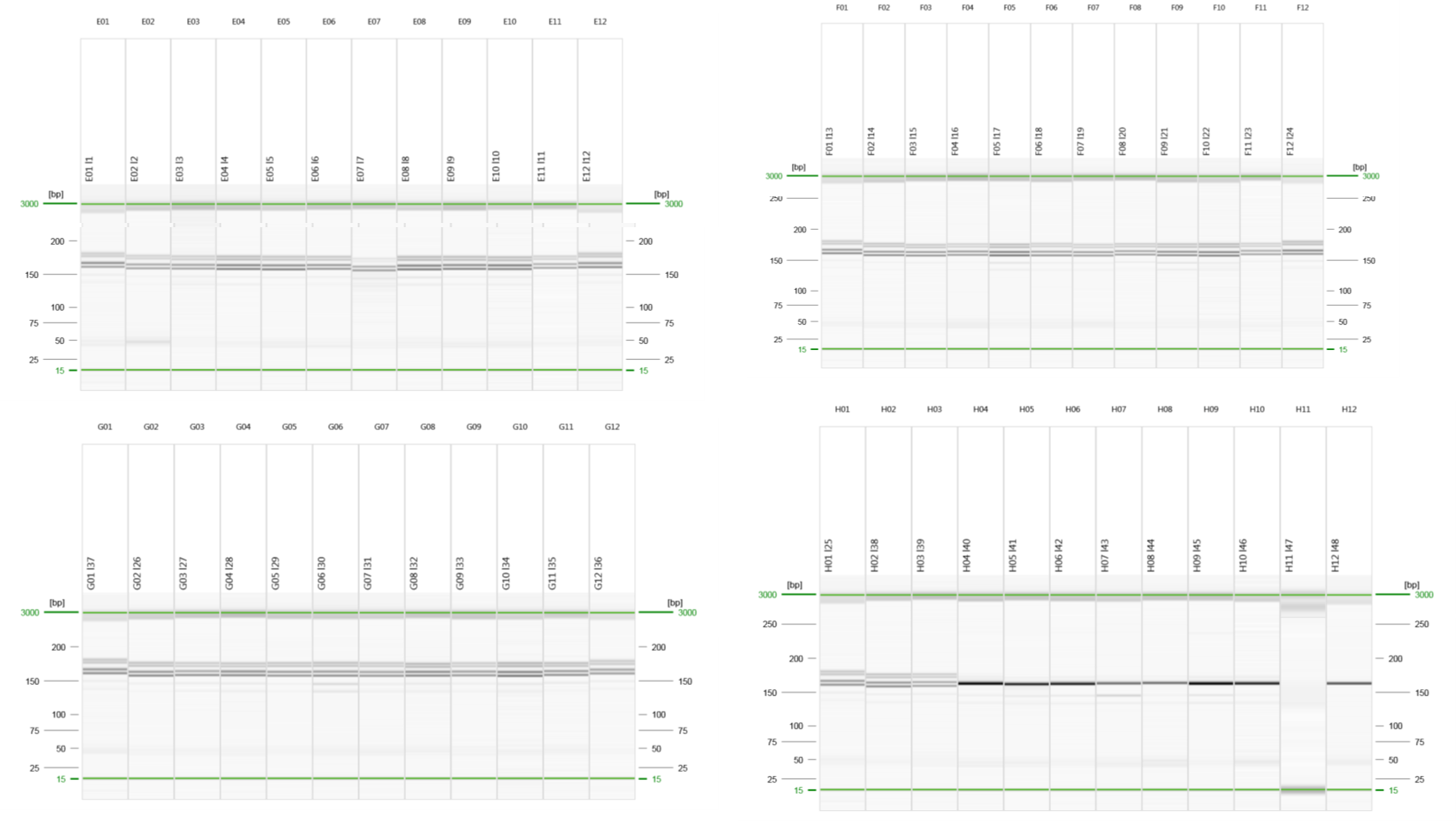

Supplement: S2 Fig — Gel image of PCR amplification of SSR marker ZOSSR91 with highest alleles on 48 germplasm accessions of ginger as captured on QIAxcel ScreenGel software. (TIF) [file pone.0259146.s002.tif]
